# Current Treatment Paradigms for Advanced Melanoma with Brain Metastases

**DOI:** 10.3390/ijms26083828

**Published:** 2025-04-18

**Authors:** Elisabetta Bonzano, Stefania Barruscotti, Silvia Chiellino, Benedetta Montagna, Chiara Bonzano, Ilaria Imarisio, Sara Colombo, Francesco Guerrini, Jessica Saddi, Salvatore La Mattina, Carlo Francesco Tomasini, Giannantonio Spena, Paolo Pedrazzoli, Andrea Lancia

**Affiliations:** 1 Department of Radiation Oncology, Fondazione IRCCS Policlinico San Matteo, 27100 Pavia, Italys.lamattina@smatteo.pv.it (S.L.M.);; 2 Dermatology Clinic, Fondazione IRCCS Policlinico San Matteo, 27100 Pavia, Italy; 3 Unit of Oncology, Department of Oncology, Fondazione IRCCS Policlinico San Matteo, 27100 Pavia, Italyb.montagna@smatteo.pv.it (B.M.);; 4 Department of Neuroscience, Rehabilitation, Ophthalmology, Genetics, Maternal and Child Health, IRCCS Ospedale Policlinico San Martino, University Eye Clinic, 16132 Genoa, Italy; 5 Unit of Neurosurgery, Department of Head & Neck Surgery, Fondazione IRCCS Policlinico San Matteo, 27100 Pavia, Italy; frague21@gmail.com (F.G.);

**Keywords:** melanoma, brain metastases, immunotherapy, targeted therapy, radiotherapy, stereotactic radiosurgery, multidisciplinary treatment

## Abstract

The therapeutic management of melanoma brain metastases has undergone a profound revolution during recent decades. Optimal integration of systemic therapies with local treatments seems to represent the strategy to pursue in order to maximize clinical outcomes, stressing the need for real multidisciplinary care in this setting of patients. However, the current approach in the clinics does not necessarily reflect what the current guidelines state, and several pending issues are present, from the ideal therapeutic sequence between stereotactic radiosurgery (SRS) and drug administration to the current role of surgery and whole brain radiotherapy (WBRT), all of which need to be addressed. This narrative review aims to provide practical help for navigating the current controversies, with an eye towards possible future advancements in the field, which could help to obtain a comprehensive molecular characterization of the tumor and a more personalized patient-centered therapeutic approach.

## 1. Introduction

Melanoma is a highly malignant tumor that develops from melanocytes; its primitive lesions, which are predominantly located in the skin, can also be found in mucous membranes and exhibit a strong inclination to aggressively metastasize to multiple organs, including the brain.

The incidence of cutaneous melanoma has been rising over the past decades, with approximately 325,000 new cases globally and over 57,000 deaths reported annually. Projections indicate that the global burden of melanoma will continue to increase, reaching an estimated 510,000 new cases and 96,000 deaths by the year 2040 [1].

The pathogenesis of melanoma involves the malignant transformation of melanocytes, typically triggered by a combination of genetic, environmental, and host-related factors. Key risk factors for primary melanoma include exposure to ultraviolet (UV) radiation, especially intermittent intense sun exposure and use of tanning beds, fair skin phenotype, the presence of multiple or dysplastic nevi, a family history of melanoma, and certain genetic mutations, such as CDKN2A or BRAF (v-Raf murine sarcoma viral oncogene homolog B1) [2].

Brain involvement unfortunately represents a common scenario in patients diagnosed with advanced melanoma; in clinical series, 30–40% of patients present brain metastases at some point during their disease course [3]; however, up to 73% of CNS involvement has been reported in autopsy series [3].

Advancements in systemic treatments, such as the use of immune checkpoint inhibitors and targeted therapies, together with improvements in radiotherapy and surgical techniques, have significantly extended survival rates for many melanoma patients. As a result, longer-term disease control has led to an increased incidence of brain metastases.

Risk factors for the development of melanoma brain metastases (MBMs) include male gender, head or neck as the primary site of disease, the presence of nodal or visceral metastases, high serum lactate dehydrogenase (LDH) levelsm and high Breslow thickness of the primary lesion [4].

Initial clinical presentation of MBMs includes headache, neurological impairment, and seizures. Around 80% of MBMs develop in the cerebral hemispheres, with a further 5% and 15% of MBMs being located in the brain stem and cerebellum, respectively [5].

The prognosis for patients with MBMs remains poor, with median survival rates often reported to be in the range from 6 to 9 months, although newer therapies have begun to improve these outcomes [6].

Early detection and accurate diagnosis are essential for tailoring the most effective treatment options and improving patient outcomes. Identifying MBMs might be challenging because the brain represents a common site for metastases from several types of primary cancers and, furthermore, symptoms can be nonspecific. Clinical presentation varies widely depending on the size, number, and location of metastases; most typical symptoms include headaches, seizures, focal neurological deficits, cognitive changes, and visual disturbances. As the disease progresses, these symptoms tend to worsen [7].

Magnetic resonance imaging (MRI) is the gold standard modality for tracking brain metastases. In addition to its high sensitivity, it provides detailed images of brain structures, and it is particularly effective in the detection of small or early metastatic lesions. Computed Tomography (CT) may be used in emergency settings when rapid imaging is required; however, CT scans tend to be less sensitive for the detection of smaller lesions, and this may lead to false negative findings. Positron Emission Tomography (PET) scans, which are used to assess the activity of cancerous cells, can also be of help in identifying metastases that may not be clearly visible with other imaging techniques [8].

In case of diagnostic uncertainty and inconclusive neuroimaging, a biopsy can be performed. Brain tissues can be obtained via proper surgical procedures or through less invasive approaches, such as stereotactic biopsy.

Tissue specimen examination is essential for differential diagnosis and melanoma confirmation. Despite sharing many histological features with primary cutaneous melanoma, MBMs show some typical traits that can help differentiate between the two of them.

MBM lesions often show infiltration of the brain parenchyma by malignant melanoma cells, typically arranged in sheets, nests, or single-file patterns. Invasive growth into surrounding tissues, including perivascular spaces, is common. This infiltrative growth pattern, often without a well-defined border, is in sharp contrast with the more localized growth of some primary brain tumors and other metastases. The presence of necrosis, hemorrhage, and inflammation is not unusual. MBMs often follow hematogenous dissemination, proving cancer aggressiveness, and biopsy specimens can show the presence of melanoma cells within blood vessels or brain parenchyma [9].

The diagnosis of MBMs can be made with the identification of malignant melanocytes that show atypical features such as large cells with pleomorphic nuclei, a high nuclear-to-cytoplasmic ratio, irregular nuclear contours, and abundant cytoplasm. The cytoplasm may contain melanin pigment, which is a hallmark of melanoma cells [10].

In cases where melanin is not visible, immunohistochemistry (IHC) is commonly used. IHC markers such as S100 protein, HMB-45, and Melan-A are specific to melanoma cells and help to differentiate them from other malignancies [10]. Additionally, SOX10 has emerged as a useful marker for identifying melanocytic tumors, including metastatic melanoma.

## 2. Methodology

This narrative review aims to summarize and critically evaluate the current evidence on the treatment of melanoma brain metastases, with a specific focus on the latest therapeutic landscape, including systemic therapies, radiotherapy, and surgical approaches. The methodology employed a structured and transparent search strategy to enhance the reproducibility and reliability of the findings. A comprehensive literature search was conducted using three major electronic databases: PubMed/MEDLINE, Scopus, and Web of Science. The search included publications available up to January 2025. Although no language restrictions were applied during the initial search phase, only studies published in the English language were ultimately included in the review.

The literature screening and selection process was conducted independently by two reviewers. Any discrepancies were resolved through discussion and consensus. Data from the included studies were extracted and synthesized narratively, highlighting key findings, treatment outcomes, and emerging trends in the management of MBMs.

## 3. Systemic Therapy

The treatment of metastatic melanoma may represent a challenging scenario in patients with clinically relevant metastatic sites, especially in the case of brain involvement. Nowadays, multidisciplinary management is key: choosing the best therapeutic approach can have a profound impact on patients’ outcomes [11].

Since 2011, the advent of novel systemic strategies, such as immunotherapy (IO) and targeted therapy (TT), has changed the natural history of metastatic melanoma. Before the availability of these drugs, first-line chemotherapy containing dacarbazine, alone or in combination with other chemotherapeutics, had shown very limited activity, with progression-free survival (PFS) rates of less than 2 months and only 25% of patients alive at 1 year; furthermore, no drug has shown activity in subsequent lines [12,13].

Currently, available medications for the treatment of metastatic melanoma have the ambition of making cancer a chronic disease. The therapeutic approach usually ranges from single-agent schedules (monotherapy) to combination therapies, with the possibility of integrating locoregional treatments without increasing toxicity and rather improving patients’ prognosis.

### 3.1. Immunotherapy

The first IO agent approved for the treatment of metastatic melanoma was ipilimumab. This anti-CTLA4 monoclonal antibody was approved by the FDA in 2011 and showed a survival advantage when compared with standard therapy [14,15]. Subsequently, two other immune checkpoint inhibitors targeting PD-1 received approval as first-line treatment options. In 2015, the use of Pembrolizumab demonstrated improved overall survival (OS) and PFS when compared with ipilimumab; furthermore, the safety profile was in favor of the anti-PD1 molecule [16]. At the same time, nivolumab showed comparable efficacy in the same setting of patients, and this led to its approval by the FDA [17].

The next effort in immunotherapy was to combine the two strategies, anti-CTLA-4 and anti-PD1, with the aim of further increasing the therapeutic outcome. Wolchok et al. demonstrated that the combination achieved the best median OS of approximately six years [18].

More recently, a new immunotherapy combination was approved: the association of nivolumab with Relatlimab (an inhibitor of lymphocyte-activation gene 3—LAG-3) demonstrated an advantage in terms of PFS when compared with nivolumab alone; furthermore, this combination showed a better safety profile than the ipilimumab/nivolumab combination. An updated analysis of the RELATIVITY-047 trial, with 3 years of follow up, confirmed that a fixed-dose combination of nivolumab and Relatlimab over nivolumab alone demonstrated continued improvements in PFS, OS, ORR, and melanoma-specific survival, supporting the long-term efficacy and safety of NIVO + RELA as a treatment option for advanced melanoma [19].

### 3.2. Targeted Therapy

The BRAF gene mutation (the most common is V600E) can be found in almost 50% of melanoma cases and represents a relevant therapeutic target. BRAF and MEK (mitogen-activated protein kinase enzyme) inhibitors (BRAFi and MEKi) block the uncontrolled proliferation mechanism permanently activated in BRAF-mutated cells; in addition, the combination of the two molecules prevents resistance and reduces toxicity.

Vemurafenib/Cobimetinib, dabrafenib/trametinib, and Encorafenib/Binimetinib are examples of BRAFi/MEKi combinations. These treatment schedules showed—when compared to standard treatments or BRAFi monotherapy—an advantage in terms of the response rate (RR), PFS, and OS [20,21,22].

Targeted therapy (TT) is generally associated with a more rapid clinical response, so it can be particularly useful in symptomatic patients, leading to tumor shrinkage in a relatively short time interval [23]. However, this transient effect is generally followed by subsequent tumor outgrowth due to the occurrence of acquired resistance, whose mechanisms are still not fully elucidated. A reactivation of mitogen-activated protein kinase (MAPK) or the utilization of an alternative pathway may be involved, with the development of resistance to TT depending, ultimately, on the incidence of multiple mutations. The recognition of this temporary effect of TT paved the way for the analysis of combination strategies, potentially reaching longer-term responses.

### 3.3. Treatment Combination and Sequencing

No head-to-head comparison studies have been conducted between immunotherapy and molecularly targeted therapy in the first-line setting; nonetheless, several trials have explored and compared different therapeutic sequences.

The DREAMseq trial randomized patients with BRAF–mutant melanoma to receive either first-line ipilimumab plus nivolumab followed by dabrafenib plus trametinib upon progression, or the reverse sequence. A total of 265 patients with BRAF-mutated metastatic melanoma were randomized, and the 2-year overall survival rate was significantly higher in the group receiving first-line immunotherapy with nivolumab/ipilimumab (72%) compared to the group receiving targeted therapy with dabrafenib/trametinib (52%). The study demonstrated that initiating treatment with the immune checkpoint inhibitor combination significantly improved overall survival compared to starting with the targeted therapy combination [24].

The phase 2 SECOMBIT trial randomized BRAF–mutant patients into three treatment arms corresponding to three different sequencing strategies. In arm A, patients received Encorafenib/Binimetinib as the first line followed by Ipi/Nivo as the second line; in arm B, patients received first-line Ipi/Nivo and Encorafenib/Binimetinib as second-line therapy; while in arm C, patients received induction therapy with Encorafenib/Binimetinib for 8 weeks followed by Ipi/Nivo, and—at disease progression—Encorafenib/Binimetinib. A total of 209 patients were enrolled in the study; the 3-year OS rate was highest (60%) in the group receiving a “sandwich” regimen (brief targeted therapy followed by immunotherapy), suggesting a potential benefit of early immunological priming. The trial showed that COMBO immunotherapy as the first line, preceded or not by induction treatment with TT, offered the best outcome in terms of OS. TT confirmed its efficacy in terms of RR and remains a valid second-line strategy. At a median follow up of 56 months, the 60-month brain metastasis-free survival rates were 56% for arm A, 80% for arm B, and 85% for arm C [25].

Adding BRAFi/MEKi to immunotherapy was, therefore, tested with the aim of obtaining both a rapid response and long-term disease control. Three combination triplets were tested: Atezolizumab/Vemurafenib/Cobimetinib, Pembrolizumab/dabrafenib/trametinib, and Spartalizumab/dabrafenib/trametinib; only the first one received FDA approval in 2020, showing a statistically significant advantage in PFS. However, the real clinical benefit of a triple combination strategy is still controversial and limited by the toxicity profile [26].

A retrospective study analyzed 683 patients with advanced BRAF-mutated melanoma treated with first-line immunotherapy or targeted therapy; the authors evaluated brain metastasis-free survival, OS, incidence of brain metastases, and sequencing strategies. The use of immunotherapy demonstrated significantly longer BMFS and OS, along with a reduced incidence of brain metastases, compared to first-line targeted therapy [27].

Another real-life study evaluated the efficacy of combined immune checkpoint inhibitors (Combi-ICI) and targeted therapy (Combi-TT) as first-line treatments for melanoma brain metastases (MBMs), finding that Combi-ICI showed significantly prolonged overall survival (OS) and progression-free survival (PFS) with sustainable intracranial and extracranial responses, whereas Combi-TT demonstrated initially higher response rates but lower durability at 12 months, with Combi-ICI proving superior in BRAFV600 patients [28].

The long-term follow up of the ABC trial demonstrates that the combination of ipilimumab plus nivolumab maintains substantial efficacy over 7 years in patients with melanoma brain metastases, highlighting the importance of this regimen as the standard of care for patients with active, asymptomatic brain metastases, with significantly improved intracranial progression-free survival and overall survival compared to nivolumab alone [29].

As a general rule, the choice of the first line treatment and subsequent schedules to be adopted is still challenging, and it has to be tailored for each patient, considering several factors, both patient related (age, comorbidities, symptoms) and disease related (presence of brain metastasis, presence of visceral disease with impaired function, possibility of locoregional approaches); furthermore, clinicians need to also consider possible previous treatments eventually received in different settings (adjuvant or neoadjuvant) when therapy for advanced disease is undertaken [30].

## 4. Local Therapy

Standard brain-directed therapies, such as neurosurgical resection, stereotactic radiosurgery (SRS), and whole brain radiation therapy (WBRT), are commonly employed to manage brain metastases (BMs). However, tumor heterogeneity and the complex scenario of the tumor microenvironment (TME) may often hinder their efficacy [31].

Determining the optimal treatment approach for brain metastases remains among the most debated issues. Local therapy selection requires careful consideration of several factors [32], including (a) the size, number, and location of the lesions, (b) the presence or absence of neurological symptoms, (c) the status of extracranial disease, expected survival, patient age, and performance status, (d) prior treatment history, (e) potential treatment-related toxicities, and, (f) finally, the expected efficacy of systemic therapies. Ongoing advancements in radiation therapy have introduced novel treatment options, leading to improved clinical outcomes for patients with melanoma brain metastases. However, the therapeutic management of brain metastases remains challenging and demands a multidisciplinary approach [33].

### 4.1. The Role of Surgery

Significant advancements in surgical techniques have revolutionized the invasive approach to brain metastases, including those from melanoma (MBMs). Procedures such as awake craniotomy, functional monitoring, and intraoperative magnetic resonance imaging (MRI) have been pivotal in improving gross total resection rates while minimizing surgical morbidity. Historically, surgery has been a cornerstone of MBM treatment, particularly in patients with a limited number of lesions, symptomatic mass effects, or those requiring histological diagnosis due to diagnostic uncertainty [32].

Surgical resection remains crucial for patients presenting with large, symptomatic tumors unresponsive to supportive care or lesions deemed unsuitable for stereotactic radiosurgery (SRS). It provides both therapeutic and diagnostic benefits, especially when other intracranial pathologies need to be excluded. Modern technologies, including neuro-navigation, fluorescence-guided surgery, and intraoperative MRI, have enhanced the precision and safety of resections, even in proximity to eloquent brain regions [34].

Optimal patient selection is essential, as surgery is most effective in individuals with controlled extracranial disease and good performance status (e.g., Karnofsky Performance Score > 70).

In patients with poor prognoses, the role of surgery may shift toward palliation, focusing on symptom relief rather than survival extension. For example, surgical intervention can rapidly alleviate severe symptoms caused by mass effects or intracranial pressure when other measures fail. It is recommended in cases of CNS metastasis-associated hemorrhage, a condition more frequently observed in melanoma brain metastases [34].

However, its role is limited by the localization of lesions, particularly in eloquent brain regions, and the potential risks of intra- and post-operative complications.

Although surgery historically represents a fundamental approach to the therapeutic management of MBMs, it generally requires adjuvant treatments, such as radiotherapy. Clinical evidence underscores the importance of surgery in improving survival outcomes when combined with other treatments. For instance, retrospective data by Fife et al. demonstrated superior survival among patients undergoing surgery or surgery plus radiotherapy compared to non-surgical management (8.9 and 8.7 months for surgery and surgery + RT vs. 3.4 and 2.1 months for RT alone and supportive care, respectively) [35].

Moreover, randomized trials have shown that post-operative radiotherapy, such as whole brain radiotherapy (WBRT), significantly reduces local recurrence rates, intracranial progression, and neurological mortality. Patchell et al. reported recurrence rates of 18% with surgery plus radiotherapy versus 70% for surgery alone [36]. These data are in line with Redmond et al. [37] and underscore the pivotal role of RT in addressing microscopic disease, improving local control, and preventing intracranial progression.

### 4.2. The Role of Radiotherapy: From WBRT to SRS

Radiotherapy (RT) is a key component of the multidisciplinary management of melanoma brain metastases, bridging local control of disease with systemic strategies.

Whole brain radiotherapy has been traditionally the standard approach for achieving comprehensive intracranial control and targeting visible metastases and microscopic disease. It has been widely regarded as the primary treatment modality for patients with multiple melanoma brain metastases [33].

WBRT, both in the post-operative setting and after previous SRS, has been a standard approach for decades. The most commonly adopted dose–fractionation regimens were 30 Gy in 10 fractions or 35 Gy in 14 fractions [38].

However, over the years, it has become evident that the use of WBRT is linked with significant neurocognitive decline, especially in memory and executive function, verbal fluency deterioration, fine motor skills, immediate recall, and delayed recall [39].

This issue has led to the development of Hippocampal Avoidance Radiotherapy (HA-RT) for the treatment of brain metastases; this technique has shown prolonged preservation of cognitive function when delivered together with memantine administration, without a detrimental effect on survival outcomes and toxicity, as described by Gondi et al. [40,41].

Given the radioresistant nature of melanoma, WBRT has demonstrated particularly limited efficacy in MBM treatment, with median survival ranging from 3 to 6 months in the absence of concurrent systemic therapy [42,43].

According to the recommendations from several international guidelines (Table 1), there is currently no clear evidence to support the use of WBRT in combination with modern systemic therapies, particularly immune checkpoint inhibitors; however, it may be considered for patients whose brain metastases have progressed during systemic therapy and who are not suitable for further surgery or SRS.

### 4.3. Stereotactic Radiosurgery

Over the past several decades, the use of stereotactic radiosurgery (SRS) has spread worldwide, and it has become the most frequently employed localized treatment for metastatic brain tumors [49] (Table 2).

Its safety and efficacy in terms of local control and survival were also tested in the clinical scenario of MBM patients [33,50]. Of note, SRS has emerged as the preferred radiotherapy modality for patients with a limited number of metastases (typically ≤5), delivering greatly conformal high-dose radiation to target lesions while sparing healthy surrounding brain tissue. Since SRS can be completed in as few as one to five sessions, it avoids delays in systemic therapy [51,52].

Advances in imaging, particularly through earlier detection of intracranial metastatic lesions via MRI, have further expanded the application of SRS, as smaller lesions identified earlier in the disease course tend to respond more favorably to this approach [51].

Concerning the post-operative setting, randomized controlled trials (RCTs) have demonstrated that applying SRS to the resection cavity significantly reduces the local recurrence rate compared to cases where no adjuvant treatment is administered after surgery [53]. Moreover, post-operative SRS offers distinct advantages over WBRT, preserving cognitive function without compromising overall survival duration [54,55].

Pedersen et al. [56] recently showed data from a nationwide study involving 838 unselected patients with MBMs (2015–2022); of these, 230 patients underwent brain metastasis surgical excision, and 30 received post-operative SRS; no significant difference in OS, intracranial PFS, or local control rates was demonstrated between patients who received post-operative SRS and those who did not.

Other studies have consistently demonstrated that SRS achieves superior local control rates, often exceeding 80% at 12 months, while significantly reducing cognitive and quality-of-life impairments compared to WBRT [57].

Brown et al. [54] conducted a randomized, controlled phase 3 trial involving adult patients (18 years and older) from 48 institutions across the USA and Canada to evaluate the impact of SRS on survival and cognitive function compared to WBRT in individuals with resected brain metastases. The median overall survival was 12.2 months for SRS and 11.6 months for WBRT. The study found that SRS to the surgical cavity led to better cognitive outcomes than WBRT. Based on these findings, the authors concluded that SRS should be considered a standard of care approach as a less toxic alternative to WBRT.

A single-center, randomized, controlled phase 3 trial compared post-operative stereotactic radiosurgery to observation in patients with completely resected brain metastases [53]. The median follow up was 11.1 months. At 12 months, freedom from local recurrence was 43% in the observation group and 66% in the melanoma SRS group. This trial, which included patients who underwent surgical resection for one to three brain metastases, demonstrated that post-operative SRS to the resection cavity significantly prolonged the time to local recurrence compared to observation. Additionally, the findings confirmed that surgical resection alone is inadequate for achieving durable local control of brain metastases [53].

Resection followed by post-operative SRS presents several challenges. First, the risk of leptomeningeal disease (LMD), particularly the nodular subtype, is increased, as well as the possibility of developing radionecrosis (RN). Another issue to be considered is the variability among clinicians in defining the target volume, which can impact treatment consistency. To address these limitations, neoadjuvant SRS administered before resection has been proposed as a potential alternative approach in an international collaboration with the INTERNEO analyses, one of the largest cohorts of patients undergoing neoadjuvant and multi-fraction SRS for brain metastases (17% from melanoma) and also one with the longest median follow up. Neoadjuvant SRS showed lower LR, RN, and nLMD rates, allowing for decreased cumulative treatment time of surgery and SRS [58].

A phase III trial from the USA comparing pre-operative to post-operative SRS is currently enrolling (NCT05438212).

In a randomized trial by Yamamoto, SRS without WBRT in patients with five to ten brain metastases was shown to be non-inferior to that delivered in patients having two to four brain metastases [59]. This finding has been reinforced by a meta-analysis evaluating tumor control probabilities for various lesion sizes treated with SRS or fractionated SRS (fSRS) [60].

For tumors ≤ 20 mm in diameter, single-dose SRS at 24 Gy achieved a 1-year local control rate of 95%. In contrast, larger tumors (21–40 mm) showed better outcomes with fractionated schedules: three to five fractions delivering 27–35 Gy yielded an 80% 1-year local control rate, suggesting that fractionation may be more effective for treating larger lesions [60].

The integration of SRS into clinical practice has also been possible due to the advancements in systemic therapies, particularly immune checkpoint inhibitors (ICIs) and targeted agents, which exhibit synergistic effects with SRS in controlling melanoma brain metastases [31].

SRS-induced immunogenic cell death has been proposed to enhance the efficacy of ICIs by promoting an abscopal effect, potentially improving systemic disease control [61].

Moreover, evidence supports the concurrent or sequential use of SRS with systemic therapies to optimize therapeutic synergy, as shown in preclinical and clinical studies [62].

Despite many advantages, SRS presents some limitations. Patients with extensive metastatic disease (>10 lesions) or diffuse intracranial burden may still require WBRT or systemic therapies as their primary treatments. Nonetheless, ongoing research continues to refine the role of SRS, particularly in combination with novel systemic agents, further solidifying its place in the evolving therapeutic landscape for melanoma brain metastases.

## 5. RT+IO Combinations

Combining systemic therapy with a local treatment, namely, SRS, recently came out as a valid therapeutic option for patients with melanoma brain metastases. Several retrospective series demonstrated improved outcomes in terms of intracranial control of disease, and encouraging data concerning PFS and OS have also been described [50]. The initial success of the multimodal strategy seems to indicate a possible synergism between the two approaches; the so-called “abscopal effect”, which refers to a radiotherapy-induced anti-tumor response in unirradiated sites of disease, has been extensively reported in the literature [63], and it may represent the evidence of the augmented efficacy of immune checkpoint inhibitors (ICIs) in stage IV melanoma. More specifically, the administration of stereotactic radiotherapy as metastasis-directed therapy could enhance tumor antigen presentation to CD8 T cells and induce the release of pro-inflammatory cytokines, ultimately leading to the hyperactivation of the immune response and immunogenic cell death outside the irradiated areas [64]. Despite this intriguing rationale, the evidence that we have collected so far on the combined approach comes from retrospective series, as we currently lack data from specifically designed randomized trials, which could help us address the open issues on its safety and the optimal therapeutic sequence.

In 2022, Franklin et al. analyzed the impact of radiation therapy in a large real-world cohort of 450 patients diagnosed with melanoma brain metastases (MBMs) and receiving three different schedules of first-line systemic therapy (combined CTLA-4 and PD-1 blockade, PD1 blockade monotherapy, or BRAF+MEKTT). The authors found out that the addition of stereotactic radiotherapy led to a significantly longer median survival, irrespective of the systemic treatment received, and SRS was confirmed as an independent prognostic factor for OS in their multivariate analysis; of interest, a positive effect on OS was also demonstrated for those patients receiving conventional radiotherapy, which included post-operative RT to the tumor cavity or whole brain radiotherapy (WBRT). Concerning toxicity, radionecrosis was described in four patients who received SRS and ICIs; the timing of SRS—before or during systemic treatment—could not be included in the multivariate analysis for the limited number of cases [65].

A multicenter retrospective series conducted by Mandalà et al. demonstrated an improved OS in MBM patients receiving IPI-Nivo combination and SRS when compared to those receiving WBRT (30.5 months vs. 18.2 months), irrespective of radiation being performed in a concomitant (within two weeks from IO start/end) or sequential manner; furthermore, the authors demonstrated a statistically significant OS benefit with the addition of SRS to immunotherapy COMBO rather than COMBO alone, both in asymptomatic and symptomatic patients [66].

The association of SRS with combination immunotherapy proved to be superior to exclusive SRS in the group of MBM patients included in the meta-analysis by Badrigilan et al.; in this study, the benefit of SRS was greater when radiotherapy was administered concomitantly with ICIs (not more than four weeks between the two treatments) rather than sequentially (ICIs prescribed more than four weeks before or after radiation) [67].

Interestingly, in another meta-analysis [68], it was shown that the co-administration of SRS with systemic therapy was associated with improved OS rather than SRS alone if only ICIs were prescribed; on the contrary, no benefit was registered in those patients receiving SRS + targeted therapy with BRAF/MEK inhibitors when compared to the ones treated with exclusive SRS.

Important data on the safety of the combined radio-immunotherapy approach can be ruled out from the pooled analysis by Congzhou Sha et al., which included nearly 14,000 patients, including MBMs, treated with ICIs alone and 1442 patients treated with ICIs + radiotherapy. The authors demonstrated comparable grade 3–4 toxicity in patients treated with ICI + RT compared to ICI alone in the setting of melanoma brain metastases, NSCLC, and prostate cancer [69].

## 6. Translational Insights into Melanoma Brain Metastases

Emerging translational research has played a crucial role in clarifying the biological mechanisms underlying melanoma brain metastases (MBMs), especially regarding resistance to immunotherapy and metastatic specificity. A key study by Mallardo et al. [70] examined the prognostic significance of the neutrophil-to-lymphocyte ratio (NLR) in patients with metastatic melanoma undergoing anti-PD-1 therapy. The findings indicated that a high baseline NLR was associated with poorer prognosis and elevated serum LDH levels. The transcriptomic analysis further demonstrated that increased NLR is linked to an immunosuppressive gene expression profile—including CCNA1, LDHA, and IL18R1—associated with inflammation and tumor progression. Conversely, a low baseline NLR corresponded with the expression of immune-activating genes such as CD3, SH2D1A, ZAP70, and CD45RA. Supporting the previous findings, Wistuba-Hamprecht et al. [71] demonstrated that the presence of CD3^+^CD45^+^ memory T cells positively correlated with favorable outcomes in stage IV melanoma patients treated with ipilimumab.

Complementary findings by Tomonobu et al. [72] unveiled a novel inhibitory mechanism involving histidine-rich glycoprotein (HRG), a plasma protein that neutralizes the pro-metastatic effects of the chemokine complex S100A8/A9, which is implicated in organotropic dissemination. In vivo models showed that HRG suppresses S100A8/A9-mediated melanoma cell migration and invasion, particularly to the brain and lungs. Notably, HRG knockdown resulted in a significant increase in brain metastases, highlighting its protective role. These results position the S100A8/A9–HRG axis as a promising therapeutic target for preventing brain metastases in melanoma.

These translational data emphasize the intricate interplay between systemic inflammation, immune suppression, and organ-specific metastatic mechanisms. Integrating these translational insights holds great promise for the development of biomarker-guided strategies and innovative therapeutic approaches aimed at improving the prevention and management of MBMs.

## 7. Conclusions and Future Perspectives

During the recent decades, the advent of immunotherapy has dramatically altered the melanoma therapeutic landscape and, in parallel, SRS seems to be on the way to fully replacing WBRT for the treatment of brain metastases due to its increased efficacy and major safety. Despite the considerable amount of retrospective data that suggests the benefit and the potential synergies of SRS with systemic therapy, currently, there is no consensus on the use of the combined treatment strategy for patients diagnosed with melanoma brain metastases [33].

Several pending issues need to be addressed, including the optimal time interval between systemic therapy start and SRS, the appropriate radiation dose to be delivered to obtain brain tumor control without impairing neurocognitive function, and, in this regard, the need for the development of novel targeted therapies and radiosensitizers that could further maximize the therapeutic index of the combination strategy [73].

Current research efforts are increasingly focused on unraveling the molecular landscape of metastatic melanoma, with the goal of identifying immune-related predictive biomarkers capable of anticipating patient response to systemic therapies or revealing mechanisms of therapeutic resistance. In the specific context of melanoma brain metastases (MBMs), there is growing interest in the complex crosstalk between tumor cells and the surrounding brain microenvironment. The identification of key signaling pathways involved in this interaction—such as those regulating immune evasion, angiogenesis, and neuroinflammation—has emerged as a promising area for the development of novel targeted therapeutic strategies [74].

A major challenge in this setting is the limited availability of tissue samples from brain lesions, which restricts direct molecular profiling and hampers the development of personalized therapeutic approaches. To overcome this limitation, artificial intelligence (AI) and machine learning (ML) tools are being explored for their ability to analyze noninvasive imaging data, such as MRI, in order to extract biologically meaningful patterns [75]. A recent study demonstrated that ML-based models could predict the brain metastasis invasion pattern (BMIP)—a histopathological biomarker associated with survival and treatment response—using only MRI-derived radiomic features. While expert radiologists achieved limited accuracy (44–59%) in predicting BMIP, the best-performing ML model reached an accuracy of 85% and an F1 score of 90%, showing strong potential for clinical application as a noninvasive, imaging-based biomarker [76].

Such integrative models, combining radiomics, radiogenomics, and deep learning approaches, may significantly enhance our ability to stratify patients, monitor treatment response, and guide therapy in MBMs, particularly when tissue sampling is not feasible. Although further validation is needed, these developments represent a major step toward precision oncology in the management of brain metastases.

Results from multiple ongoing prospective clinical trials are eagerly awaited in order to better define the standard of care in the setting of MBM patients, starting from the assumption that multidisciplinary shared decision making remains key and the proposed therapeutic approach should be patient tailored and aim to preserve patients’ quality of life.

## Figures and Tables

**Table 1:** Summary of international guidelines on the use of whole brain radiotherapy (WBRT) in melanoma brain metastases.

Organization	Recommendations	Consensus Rate/Strength
European Dermatology Forum (EDF), European Association of Dermato-Oncology (EADO), European Organization of Research and Treatment of Cancer (EORTC) [44]	Whole brain radiotherapy can no longer be recommended for the treatment of melanoma brain metastases.	100%
European Society for Medical Oncology (ESMO) consensus [45]	WBRT is not recommended after complete resection or SRS of MBMs. The routine use of WBRT in MBMs not amenable to SRS and in LMD is discouraged and should be restricted to carefully selected patients.	100%
Cancer Council Australia [46]	WBRT is used as the last-line palliative therapy for patients with multiple brain metastases that have progressed on systemic and/or local therapies.	—
US National Comprehensive Cancer Network (NCCN) [47]	In cases of disseminated systemic disease with limited treatment options, hippocampal avoidance WBRT (HA-WBRT) with memantine may be considered.	—
American Society for Radiation Oncology (ASTRO) [48]	For patients with a favorable prognosis and brain metastases not eligible for surgery or SRS, WBRT (HA-WBRT plus memantine) is recommended as a primary treatment option.	Strong recommendation

**Table 2:** Summary of international guidelines on the use of stereotactic radiosurgery (SRS) in melanoma brain metastases.

Organization	Recommendations	Consensus Rate/Strength
European Dermatology Forum (EDF), European Association of Dermato-Oncology (EADO), European Organization of Research and Treatment of Cancer (EORTC) [44]	Eligible patients with brain metastases should be treated with stereotactic radiotherapy. Surgery may be considered when SRS is not feasible.	100%
European Society for Medical Oncology (ESMO) consensus [45]	Post-operative SRS should be considered after complete resection of MBMs. SRS with concurrent immunotherapy or targeted therapy appears safe, though high-level evidence is lacking. SRS is preferred for limited asymptomatic BMs (defined as 1–4 BMs ≤ 4 cm, or 5–10 BMs ≤ 3 cm with total volume ≤ 15 mL).	97% 90%
Cancer Council Australia [46]	SRS is recommended for a single or a small number (≤3) of asymptomatic brain metastases to maximize local tumor control.	
US National Comprehensive Cancer Network (NCCN) [47]	Recommends SRS as primary treatment for patients with limited or multiple asymptomatic melanoma brain metastases.	—
American Society for Radiation Oncology (ASTRO) [48]	SRS or WBRT is strongly recommended after surgical resection to improve intracranial control. In patients with resected brain metastases and limited additional BMs, SRS is strongly recommended over WBRT.	Strong recommendation
